# Carbon-Based Catalysts for Electrochemical Nitrate Reduction to Ammonia: Design Strategies and Mechanistic Insights

**DOI:** 10.3390/ma18133019

**Published:** 2025-06-25

**Authors:** Qunyu Chen, Liuyang Deng, Jinrui Zhang, Ying Zhang, Lei Zhang, Shun Lu, Yanwei Wang

**Affiliations:** 1 School of Chemical Engineering, Xuzhou College of Industrial Technology, Xuzhou 221140, China; 2 Chongqing Institute of Green and Intelligent Technology, Chinese Academy of Sciences, No. 266, Fangzheng Avenue, Chongqing 400714, China

**Keywords:** carbon-based catalysts, electrochemical nitrate reduction, ammonia production, material design, mechanistic insights

## Abstract

The electrochemical reduction of nitrate to ammonia offers a promising solution for both alleviating nitrate pollution in wastewater and providing a sustainable ammonia source for agriculture use. This review focuses on the role of carbon-based catalysts in electrochemical nitrate reduction to ammonia, emphasizing their potential in addressing environmental pollution and supporting sustainable ammonia production. Carbon materials, known for their abundance, affordability, and eco-friendly properties, are central to this process. The review highlights key strategies for enhancing catalytic performance, including heteroatom doping, the development of porous structures, and the integration of metal/metal oxide nanoparticles. Additionally, it addresses significant challenges such as weak nitrate adsorption, slow reaction kinetics, and competition with the hydrogen evolution reaction. Through the integration of advanced material design, mechanistic insights, and innovative engineering strategies, this review provides valuable guidance for the future design of carbon-based catalysts, paving the way for significant advancements in both nitrate removal and sustainable ammonia synthesis.

## 1. Introduction

The increasing presence of nitrate pollution in water bodies has become a growing environmental concern globally [1]. Nitrate contamination originates mainly from agricultural runoff, industrial effluents, and wastewater discharges, leading to detrimental effects on ecosystems and public health [2,3]. Traditional approaches of nitrate treatment from water, such as biological denitrification and reverse osmosis, are often costly, energy-intensive, and produce undesirable by-products [4,5]. This highlights the urgent need for efficient, sustainable, and economically viable alternatives for nitrate remediation. Recently, the electrochemical nitrate reduction reaction (NO_3_RR) has emerged as a promising technology to address this challenge, not only for nitrate removal but also for the production of valuable by-products, particularly ammonia (NH_3_) [6,7,8]. NH_3_, a key component of fertilizers, has traditionally been synthesized through the energy-intensive Haber–Bosch process. Electrochemical NO_3_RR, however, offers a potential pathway for “green ammonia” production, aligning with both environmental sustainability goals and the growing demand for ammonia in agriculture.

Among the various catalytic materials explored for NO_3_RR, carbon-based catalysts have attracted considerable interest because of their unique advantages. Carbon materials are abundant, low-cost, environmentally friendly, and exhibit high electrical conductivity [9,10,11]. Moreover, their structural properties can be easily tailored to enhance their catalytic performance, making them ideal candidates for electrochemical applications [12,13]. These materials can be modified at the atomic and molecular levels, allowing for improved activity, selectivity, and stability in various reactions, including nitrate reduction. The versatility of carbon-based materials such as carbon nanotubes (CNTs), graphene, and various carbon composites further expands their applicability in NO_3_RR processes, providing ample opportunities for innovation in catalyst design and optimization.

Despite their potential, carbon-based catalysts for NO_3_RR face several challenges. The weak adsorption of nitrate ions on carbon surfaces hinders efficient reduction, as strong interactions are needed for electron transfer and reduced activation energy. Additionally, slow reaction kinetics lead to low current efficiencies. The competitive hydrogen evolution reaction (HER) further complicates the process, diverting electrons from nitrate reduction and reducing selectivity [14,15,16,17,18]. To improve efficiency, carbon-based catalysts must suppress HER and enhance nitrate reduction by optimizing their electronic structure, surface properties, and reaction conditions. Significant advancements have been achieved in addressing these challenges in recent years. Researchers have developed various strategies to improve the performance of carbon-based catalysts for NO_3_RR, such as doping with heteroatoms (e.g., nitrogen, sulfur, phosphorus) to modify the electronic properties of the carbon surface, creating porous structures to increase the surface area and improve nitrate adsorption, and designing composite catalysts by integrating carbon materials with metal or metal oxide nanoparticles to boost catalytic activity [19,20,21,22]. These strategies aim to enhance the binding affinity for nitrate ions, accelerate electron transfer, and suppress HER, thereby improving the overall performance of the electrochemical nitrate reduction process.

In this review, we provide an in-depth analysis of the design strategies and mechanistic insights related to carbon-based catalysts for NO_3_RR. We mainly highlight the unique properties of carbon-based materials in this context and outline the key challenges associated with their use. The review further explores the latest advances in catalyst design, focusing on strategies to improve nitrate adsorption, enhance catalytic kinetics, suppress HER, and improve catalyst durability. By synthesizing the current state of research, this review aims to offer valuable insights into the future direction of carbon-based catalyst development for NO_3_RR, ultimately contributing to more efficient and sustainable nitrate removal and ammonia production technologies.

## 2. Experimental Techniques for NO_3_RR

### 2.1. Fundamental Electrochemical Cell Configurations

The H-cell setup is widely employed for initial catalyst screening. This configuration consists of two compartments—an anode and a cathode—separated by an ion-exchange membrane to prevent product crossover and oxidation at the anode. The working electrode, coated with the carbon-based catalyst, is submerged in the catholyte. A counter electrode, such as a Pt wire or graphite rod, and a reference electrode, like Ag/AgCl, Hg/HgO, or SCE, complete the three-electrode system, typically controlled by a potentiostat/galvanostat. The catholyte volume is usually small, and the system is often purged with an inert gas before and during experiments to exclude oxygen. While versatile and simple, H-cells may experience significant mass transport limitations, particularly at high current densities.

Flow cells are increasingly utilized for experiments requiring higher reaction rates and enhanced mass transport. In these systems, catalyst-coated electrodes, such as gas diffusion electrodes, are incorporated into a cell where the catholyte or the anolyte is continuously circulated. This design enhances nitrate flux to the catalyst surface, enabling the study of reactions at industrially relevant current densities. Flow cells are critical for evaluating practical feasibility but introduce engineering challenges related to sealing and product separation/analysis.

### 2.2. Electrochemical Testing Protocols

Carbon materials are typically dispersed in a solvent mixture (e.g., water, isopropanol), binder (e.g., Nafion), and sometimes conductive agents (e.g., carbon black). The resulting ink is drop-cast, spray-coated, or doctor-bladed onto conductive substrates such as carbon paper, carbon cloth, glassy carbon, or Ti foil.

Cyclic voltammetry is used to probe the redox behavior of the catalyst, estimate the electrochemical active surface area, and identify the onset potentials for nitrate reduction and associated reaction kinetics.

Linear sweep voltammetry provides steady-state or pseudo-steady-state current measurements as a function of applied potential, generating polarization curves to assess the activity of different catalysts.

Chronoamperometry (CA) and chronopotentiometry (CP) are employed to quantify reaction products over extended periods. In CA, a fixed potential is applied, while in CP, a constant current density is used. Both methods typically run for 1–2 h for initial screening and longer for stability tests. The electrolyte is sampled periodically for product analysis and yield quantification.

### 2.3. Key Performance Metrics

The ammonia yield rate is the mass of NH_3_ produced per unit time and per unit geometric area of the electrode (e.g., mg h^−1^ cm^−2^) or per unit mass of catalyst (e.g., mg h^−1^ mgcat^−1^).

Faradaic efficiency (FE_NH3_) is the percentage of the total charge passed that is utilized for ammonia production. It is calculated as:

FE_NH3_ = (n·F·C_NH3_·V/Q·M) × 100%

where n is the number of electrons transferred per NH_3_ molecule (8e^−^), F is Faraday’s constant, C_NH3_ is the ammonia concentration, V is the electrolyte volume, Q is the total charge passed, and M is the molecular weight of NH_3_.

The selectivity of the product is defined as the ratio of the concentration of the desired product to the degraded concentration of nitrate, expressed as a percentage.

Stability and selectivity over time are assessed through long-term CA/CP experiments with periodic product analysis. Post-mortem catalyst characterization is often conducted to determine the stability and selectivity of the catalyst over extended reaction times.

## 3. Carbon Material Categories for NO_3_RR

The selection and design of carbon-based materials are crucial for improving the efficiency of NO_3_RR. These materials can be broadly classified into two main categories: intrinsic carbon materials and functionalized carbon-based composites. Intrinsic carbon materials, including activated carbon, graphene, and CNTs, exhibit distinctive characteristics such as a high surface area and electrical conductivity, making them advantageous for electrochemical processes. On the other hand, functionalized carbon-based composites incorporate heteroatoms, metals, or metal oxides to enhance catalytic activity, improve nitrate adsorption, and overcome challenges like slow reaction kinetics and hydrogen evolution competition.

### 3.1. Intrinsic Carbon Materials

Intrinsic carbon materials, including graphene, CNTs, and porous carbon architectures, demonstrate critical advantages for NO_3_RR applications owing to their superior electrical conductivity, tailorable surface chemistry, and natural abundance. These intrinsic characteristics enable essential functional enhancements in electrocatalytic systems by promoting efficient electron transport mechanisms, optimizing nitrate ion adsorption kinetics, and establishing favorable mass transfer pathways throughout the reduction process.

#### 3.1.1. Graphene/Oxidized Graphene

Graphene, composed of sp^2^-hybridized carbon atoms in a hexagonal structure, is a highly efficient electron conductor, making it a perfect choice for electrochemical applications [23,24,25]. The flat, two-dimensional structure of graphene facilitates effective electron transfer during the reduction of nitrate. Graphene and graphene oxide are rarely used directly as electrocatalysts in the NO_3_RR. Instead, their catalytic performance is typically enhanced by introducing defects or modifications to their structure. These modifications can include doping with various elements (such as nitrogen, boron, or sulfur), creating vacancies, or functionalizing the surface with oxygen-containing groups. These approaches help to optimize the electronic properties and increase the catalytic activity of graphene-based materials, making them more effective for nitrate reduction reactions. Recent advancements in this field have focused on controlling the density and type of defects to improve the selectivity and efficiency of the electrochemical process, paving the way for more efficient and sustainable catalysts for NO_3_RR. The specific research progress will be discussed in the following sections.

#### 3.1.2. Carbon Nanotubes

**Figure 1:**
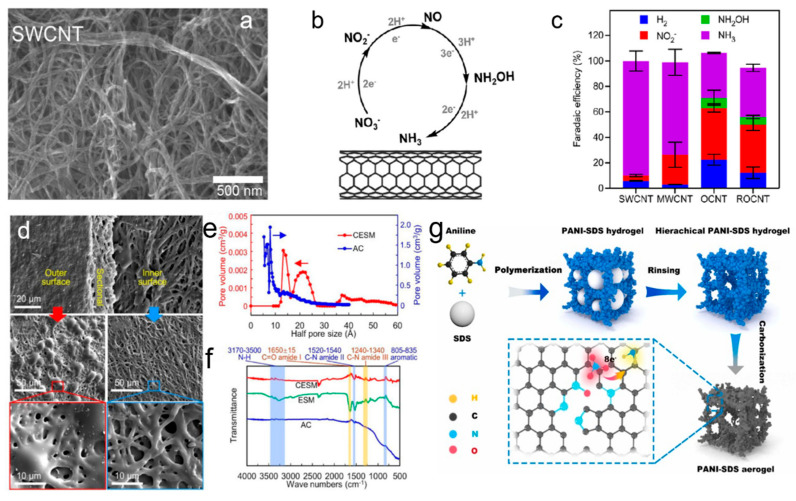
(**a**) SEM images of CNT. (**b**) Potential reaction pathway for the reduction of NO_3_^−^ to NH_3_. (**c**) Distribution of products for various CNTs at –0.85 V in a solution of 0.1 M PB + 0.4 M KNO_3_. Reproduced from ref. [28] copyright (2022), with permission from the American Chemical Society. (**d**–**f**) Characteristics of CESM and AC: (**d**) SEM images; (**e**) pore size distribution for different carbon materials; (**f**) FTIR spectra for various carbon materials. Reproduced from ref. [29] copyright (2025), with permission from Elsevier. (**g**) Schematic of the synthesis process for N-C-1000 catalysts derived from hydrogels. Reproduced from ref. [30] copyright (2025), with permission from Elsevier.

CNTs are another promising class of carbon-based materials for NO_3_RR. Their one-dimensional tubular structure induces a confinement effect, which can modulate reaction pathways at the atomic level. CNTs provide high surface area and structural integrity, which are crucial for stabilizing the active sites involved in nitrate reduction [26,27]. The unique confinement effect helps control the adsorption of nitrate ions, thereby guiding the reaction toward the desired product. Additionally, CNTs exhibit excellent conductivity, which further enhances the overall electrochemical performance of the catalyst. The researchers discovered that pristine multi-walled carbon nanotubes (MWCNTs) displayed significant electrocatalytic performance for the reduction of NO_3_^−^ to NH_3_ in a near-neutral solution, while single-walled CNTs showed even greater activity and selectivity (Figure 1a–c). Different from the conventional belief that heteroatom doping improves electrocatalytic performance, the introduction of oxygen and nitrogen groups to MWCNTs actually decreased their activity for NO_3_^−^ reduction. These results suggest that carbon serves as the active site for NO_3_RR [28].

#### 3.1.3. Porous Carbon

Porous carbon materials, such as activated carbon and carbon aerogels, are known for their high surface area and tunable pore structures. These materials offer significant advantages in terms of nitrate ion enrichment and mass transport optimization. The hierarchical porous structure of these materials facilitates the diffusion of nitrate ions and provides abundant active sites for adsorption, which is critical for improving the efficiency of NO_3_RR [30,31]. Chen et al. developed an ultrathin porous electrode from eggshell bio-waste for the electrosorption of anionic species [29]. The carbonized eggshell membrane (CESM) displayed a hierarchical pore structure and, despite its low surface area, showed a significantly higher ion adsorption capacity than activated carbon (Figure 1d–f). CESM exhibited the highest selectivity for NO_3_^−^ over competing anions. Density functional theory (DFT) calculations revealed that nitrogen-containing functional groups in a porous structure played a key role in NO_3_^−^ adsorption. These findings highlighted the potential of porous biomaterials for selective NO_3_^−^ electrodes and suggested pathways for enhancing their performance through activation or functionalization.

### 3.2. Functionalized Carbon-Based Composites

Functionalization of carbon materials through heteroatom doping or the incorporation of metal catalysts has emerged as an effective strategy to enhance their catalytic performance for NO_3_RR. These modifications can significantly alter the electronic properties and surface characteristics of the carbon materials, facilitating better nitrate adsorption and electron transfer while suppressing unwanted reactions like HER.

#### 3.2.1. Heteroatom-Doped Carbon

**Figure 2:**
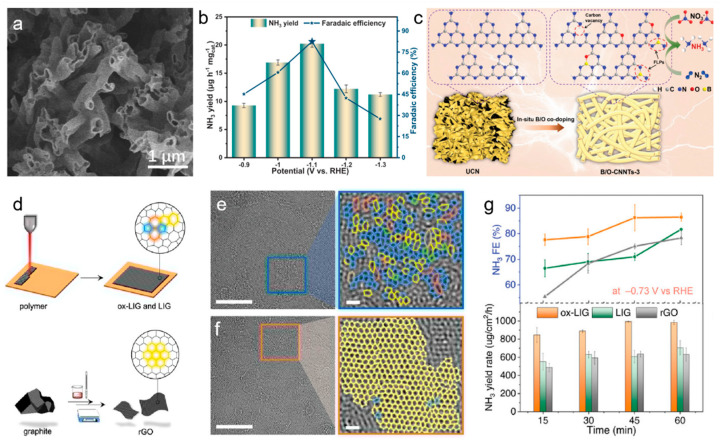
(**a**) SEM image of B/O-CNNTs. (**b**) The NH_3_ production yield and FE for NO_3_RR. (**c**) Schematic illustration for the transformation of carbon nitride to B/O-CNNTs for NO_3_RR reactions. Reproduced from ref. [37] copyright (2024), with permission from Wiley-VCH. (**d**) Schematic of the preparation of GO-based catalysts. HRTEM images of ox-LIG (**e**) and rGO (**f**). (**g**) Time-dependent FE and yield rate of NH_3_ at –0.73 V vs. RHE. Reproduced from ref. [40] copyright (2023), with permission from Wiley-VCH.

Doping carbon materials with heteroatoms such as nitrogen (N), boron (B), phosphorus (P), and sulfur (S) introduces functional groups that modify the electronic structure and enhance the reactivity of the material. N doping, in particular, has been widely studied for its ability to induce charge redistribution and create more favorable sites for nitrate adsorption. For example, pyridinic N sites are known to promote the adsorption of NO_3_^−^, while graphitic N can enhance conductivity and improve the overall electrochemical performance (Figure 1g) [30,32,33]. B and P doping can disrupt the inherent inertness of carbon through activating the surface and enabling more effective stabilization of intermediate species like nitrite [34,35,36]. In Jiang et al.’s study on B and O dual-doped carbon nitride nanotubes (B/O-CNNTs) synthesized via a copolymerization process, the dual doping of B and O not only altered the coordination environment and electronic structure of the carbon nitride framework, but also introduced frustrated Lewis pairs, which were crucial for activating chemisorbed nitrate (Figure 2a–c). The B/O-CNNTs achieved maximum Faradaic efficiencies of 96% at −1.1 V vs. RHE for NO_3_RR, with a corresponding ammonia yield of 211.4 μg h^−1^ mg^−1^. The unique 1D nanotubular structure, carbon vacancies, and the synergistic effect of B/O doping enhanced interactions among reactants and boosted electron transfer rates [37]. These modifications helped to break the carbon material’s inertness and optimized its catalytic properties for NO_3_RR. Heteroatom-doped carbon materials are commonly utilized as catalyst supports, as the incorporation of heteroatoms alongside metal species creates a coordinated environment that can modulate the d-band structure of metal. This interaction effectively fine-tunes the electronic properties of the metal, enhancing catalytic performance.

#### 3.2.2. Defective Carbon

The introduction of defects in carbon materials, such as vacancies or edge defects, is another promising approach to enhance catalytic performance. These defects create active sites that can serve as favorable adsorption points for nitrate ions and facilitate electron transfer during the reduction reaction. Vacancies, such as five-membered or seven-membered rings, are particularly useful for enhancing catalytic activity in electrochemical reactions by providing a high density of reactive sites. Surface functional groups, like -COOH or -OH, also play a crucial role in tuning the hydrophilicity of the material, which can influence the adsorption behavior of nitrate and improve mass transport [38,39].

Huang et al. explored amorphous graphene with structural strains and disorders synthesized by laser induction for nitrate reduction, revealing that its unique carbon lattice and oxygen-containing groups enhanced catalytic activity for nitrate-to-ammonia conversion (Figure 2d–g) [40]. The amorphous graphene exhibited a Faradaic efficiency of approximately 100% and an ammonia production rate of 2859 μg cm^−2^ h^−1^ at −0.93 V vs. the RHE. The researchers used X-ray pair-distribution function analysis and electron microscopy to uncover the material’s molecular features that facilitate nitrate reduction. In situ Fourier transform infrared spectroscopy and theoretical calculations highlighted the role of these features in stabilizing reaction intermediates through structural relaxation. This work demonstrated the potential of defective carbon materials in electrochemical nitrate reduction for applications like wastewater remediation and sustainable agricultural practices. Du et al. developed a range of carbon-based catalysts with tailored quaternary-N and N vacancies, emphasizing the importance of dual defect sites in improving the NO_3_RR process [15]. The NHC-1000 catalyst, with precisely engineered active sites, achieved a high NH_3_ Faradaic efficiency of 91.2% and a NH_3_ yield rate of 2.6 mmol h^−1^ g^−1^ at −0.5 V (vs. RHE). Structural characterization and theoretical studies revealed that the quaternary N played a key role in the rate-determining step of *NO protonation to *NHO and aided in the formation of *NH_2_ intermediates through the combined action of N vacancies.

#### 3.2.3. Carbon-Supported Metal Catalysts

The incorporation of metal-based nanoparticles or single atoms into carbon supports significantly improves both the catalytic activity and stability of electrocatalysts. The metal active sites, which are well-dispersed on the carbon support, benefit from the high surface area, tunable electronic properties, and conductive nature of carbon materials [41,42,43]. This synergistic interaction not only facilitates efficient electron transfer during the nitrate reduction reaction, but also ensures the long-term durability of the catalyst. Moreover, the carbon support can effectively stabilize the metal species, preventing aggregation and enhancing the overall performance. This approach not only boosts the efficiency of nitrate reduction, but also provides opportunities for precise control over the reaction pathways, improving selectivity and minimizing unwanted side reactions.

**Figure 3:**
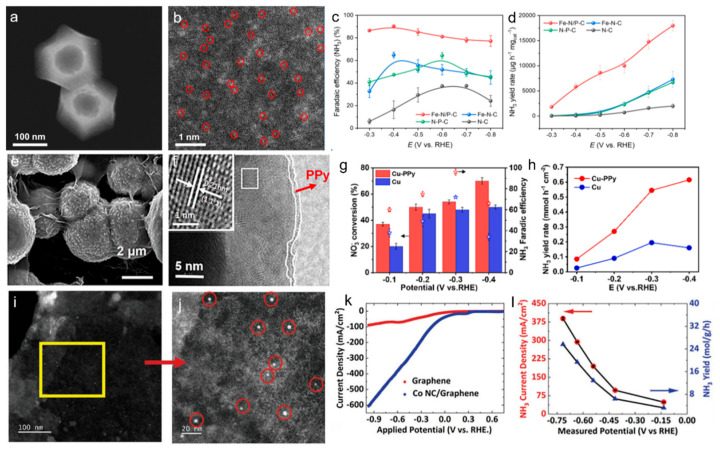
(**a**) HAADF-STEM image and (**b**) aberration-corrected HAADF-STEM of Fe-N/P-C. (**c**) Potential-dependent FE of NH_3_ and (**d**) the corresponding NH_3_ production rate over four catalysts for 0.5 h. Reproduced from ref. [44] Copyright (2023), with permission from Wiley-VCH. (**e**) SEM image of Cu-PPy. (**f**) HRTEM images of Cu-PPy. (**g**) NO_3_^−^ conversion and NH_3_ FE of Cu-PPy and Cu at different applied potentials over 2 h. (**h**) NH_3_ yield rate for Cu-PPy and Cu at various applied potentials for 2 h. Reproduced from ref. [45] copyright (2025), with permission from Elsevier. (**i**) Characterization of Co NC/graphene catalyst, (**j**) STEM images of CoO NC/graphene at increasing magnifications. (**k**) LSV curves for graphene and CoO NC/graphene in 1 m KOH + 1 m KNO_3_ electrolyte. (**l**) NH_3_ current densities, yield, and measured potentials during chronopotentiometry at constant current densities −400, −300, −200, −100, and −50 mA cm^−2^. Reproduced from ref. [47] copyright (2023), with permission from Wiley-VCH.

For instance, this is demonstrated in an Fe single-atom catalyst coordinated with N and P on a hollow carbon polyhedron [44]. The introduction of P atoms disrupted the intrinsic charge symmetry surrounding the Fe-atom catalyst, improving the adsorption of NO_3_^−^ and promoting the accumulation of crucial reaction intermediates. The metal atoms provided highly active sites that facilitate electron transfer and stabilize reaction intermediates, thus boosting the efficiency of the reduction reaction. This catalyst achieved 90.3% NH_3_ Faradaic efficiency and a yield rate of 17,980 μg h^−1^ mgcat^−1^ (Figure 3a–d). Moreover, a conductive polymer protection approach was used by modifying polypyrrole onto Cu nanoparticles (Cu-PPy), resulting in enhanced NO_3_^−^ reduction efficiency under strongly acidic electrolyte [45]. In 0.5 M H_2_SO_4_, the Cu-PPy catalyst delivered an NH_3_ yield rate of 0.55 mmol h^−1^ cm^−2^ with 99.99% NH_3_ selectivity and 96.0% Faradaic efficiency at high NO_3_^−^ conversion. The PPy layer protected Cu from corrosion, promoted NO_3_^−^ and NO_2_^−^ adsorption, stabilized Cu in an intermediate valence state, and accelerated electron transfer (Figure 3e–h). Furthermore, the study constructed a co-production system of NH_3_ and adipic acid, achieving a high combined electron efficiency.

Carbon materials are also crucial in supporting metal alloys and their oxides for NO_3_RR. The combination of carbon with metal alloys or oxides offers a unique synergy that enhances both catalytic performance and stability. The high surface area and electrical conductivity of carbon provide an ideal environment for the efficient dispersion of metal species, ensuring improved electron transfer during the reaction. Metal alloys, with their tunable electronic properties, can effectively modulate the adsorption and activation of NO_3_^−^, while metal oxides can offer enhanced stability and resistance to corrosion. For instance, RuCo alloy nanosheets on pinewood-derived three-dimensional porous carbon (RuCo@TDC) were developed via electrodeposition [46]. This catalyst showed superior electrocatalytic performance, achieving an ammonia yield of 2.02 ± 0.11 mmol h^−1^ cm^−2^ at −0.6 V and a Faradaic efficiency of 95.7 ± 0.8% at −0.2 V in 0.1 M KOH and 0.1 M KNO_3_ electrolyte. The outstanding performance of RuCo@TDC was due to the synergistic effect between Ru and Co, as well as the favorable three-dimensional porous nanostructure of carbon support. In addition, Kani et al. reported CoO nanoclusters modified on graphene as an effective catalyst for NO_3_RR [47]. The catalyst showed exceptional performance with over 98% NH_3_ Faradaic efficiency and a high NH_3_ current density of approximately 400 mA cm^−2^. The high mass activity and selectivity of the CoO nanoclusters on graphene made it a promising catalyst for sustainable ammonia production with reduced energy consumption and enhanced efficiency. Furthermore, graphene served as an excellent support material due to its high electrical conductivity and large surface area, which facilitated electron transfer and provided ample space for the active CoO nanoclusters (Figure 3i–l).

Table 1 presents a critical trade-off analysis of various carbon material platforms for NO_3_RR. It compares key factors such as structural characteristics, electrochemical performance, stability, and selectivity across different carbon-based materials. This table serves as a comprehensive overview of the strengths and limitations of each material, providing valuable insights for optimizing catalyst design and improving the efficiency of the NO_3_RR process.

## 4. Mechanistic Insights

### 4.1. Adsorption and Activation of Reactive Species

The porous structure of carbon materials provides a large surface area, promoting better contact between the nitrate ions and the catalyst surface. Surface modifications, such as doping with heteroatoms (e.g., nitrogen, sulfur) or functional group introduction, can improve the adsorption affinity and the electron transfer efficiency. Additionally, tailoring the electronic structure of carbon materials can optimize the activation of reaction intermediates, facilitating the reduction process. These strategies collectively enhance the catalytic activity, selectivity, and stability of carbon-based catalysts for nitrate electroreduction to ammonia.

**Figure 4:**
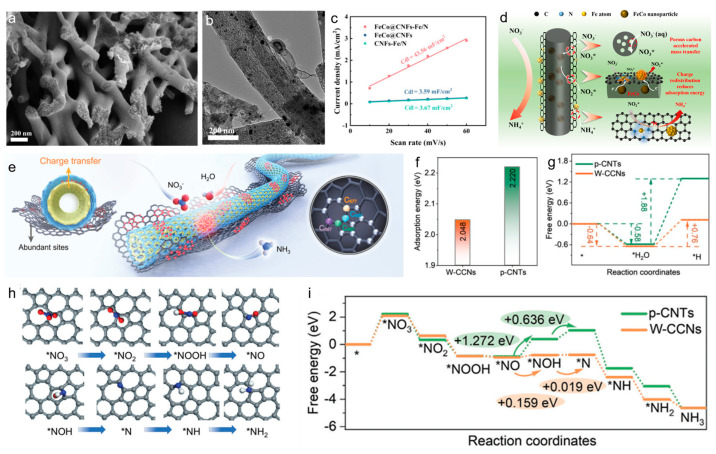
(**a**) SEM and (**b**) TEM images of FeCo@CNFs-Fe/N. (**c**) The C_dl_ of different prepared catalysts. (**d**) Mechanism illustration of electrocatalytic NO_3_RR on FeCo@CNFs-Fe/N. Reproduced from ref. [51] copyright (2025), with permission from Elsevier. (**e**) Schematic diagram of W-CCNs and the potential active sites for NO_3_RR. (**f**) The comparison of adsorption energy of *NO_3_ on W-CCNs and p-CNTs. (**g**) The reaction energies of *H formation over W-CCNs and p-CNTs through H_2_O electrolysis. (**h**) Adsorption configurations of NO_3_RR intermediates on W-CCNs (C: gray, N: blue, O: red, H: light white). (**i**) Free energy diagrams for NH_3_ formation from NO_3_RR on p-CNTs and W-CCNs without pH correction. Reproduced from ref. [57] copyright (2025), with permission from Wiley-VCH.

The porous structure of carbon facilitates mass transfer and the adsorption of reactive species, providing essential conditions for further activation [48,49,50]. Chen et al. present a novel Fe/N active site-functionalized porous carbon nanofiber (CNF) catalyst with encapsulated FeCo nanoparticles (FeCo@CNFs-Fe/N) for NO_3_RR [51]. The FeCo@CNFs-Fe/N electrocatalyst demonstrated excellent performance, attaining an NH_3_ yield of 498.18 μmol/(h g_cat_) and a notable reduction in overpotential by 0.565 V. This performance surpassed that of both comparable CNF-based catalysts by 3 to 10 times (Figure 4a–d). The enhanced catalytic efficiency was largely due to the porous carbon structure, which improved mass transfer and facilitated efficient adsorption of reactive species. Furthermore, the adjusted spatial arrangement of active sites promoted faster electron transfer and lowered the reaction energy barrier. Another study focused on an atomically dispersed RuCu dual-atom catalyst (RuCu DAs/NGA) supported on nitrogen-doped graphene aerogels (NGA), synthesized via a pulsed discharge strategy [52]. This catalyst achieved high Faraday efficiency and ammonia yield, with in situ studies revealing the dynamic evolution of asymmetric RuN_2_-CuN_3_ active sites during the nitrate reduction process. Density functional theory calculations demonstrated that the Ru atom played a crucial role in the RuN_2_-CuN_3_/C structure during electrocatalytic NO_3_RR. In the early stages, Ru and Cu atoms work together to reduce *NO_3_ to *NO, while in the later stages, the Ru atom primarily reduces *NO to *NH_3_. The synergistic interaction between Ru and Cu atoms lowers the reaction free energy and optimizes the adsorption and desorption of intermediates, thus enhancing the NO_3_RR process. The nitrogen-doped graphene support also played a crucial role in dispersing and stabilizing the metal atoms, preventing their aggregation and thus maintaining the catalyst’s activity and selectivity.

Furthermore, a recent study demonstrated that doping carbon materials with nitrogen has been shown to effectively regulate the charge distribution, thereby facilitating the binding and reduction of NO_3_^−^ [53,54,55]. The Fe@N10-C catalyst, which incorporated nitrogen-doped carbon, exhibited remarkable performance in nitrate removal and ammonia production [56]. The nitrogen doping not only activated neighboring carbon atoms, but also modulated the electronic structure and chemical environment of the catalyst, enhancing the adsorption of nitrate and the overall reaction process. The heterointerface formed between the Fe nanoparticles and nitrogen-doped carbon resulted in charge rearrangement at the interface, which affected the adsorption of reactant species and boosted reaction kinetics. The results of DFT simulations indicated that the optimal nitrogen-doped catalyst (Fe@N10-C) had a more favorable adsorption of nitrate ions compared to the undoped catalyst, making it more efficient for the nitrate reduction reaction.

In addition, carbon with well-designed defects can create additional active sites, enhancing the efficiency of NO_3_RR. An example of this is the hierarchical carbon-based metal-free electrocatalyst (C-MFEC) formed by winged carbon coaxial nanocables (W-CCNs), which demonstrated functional separation properties [57]. The pristine CNTs’ inner core enabled efficient charge transfer, while the outer shell, made up of in situ-generated graphene nanosheets and carbon layers with enriched topological defects, significantly contributed to enhancing NO_3_^−^ adsorption, water dissociation, and N-H bond formation. The W-CCNs exhibited outstanding performance for NO_3_RR, operating efficiently across a wide pH range. Theoretical calculations revealed that the C577 sites of W-CCNs were more favorable for adsorbing NO_3_^−^, while the adjacent C557 sites contributed to generating active hydrogen from water dissociation (Figure 4e–g). The adsorption of *NO_3_ was identified as the rate-determining step (RDS) for both p-CNTs and W-CCNs. W-CCNs exhibited a smaller ΔG of 2.05 eV compared to p-CNTs (+2.22 eV), indicating that topological defects in W-CCNs enhance *NO_3_ adsorption. Moreover, significant hydrogenation steps occur on *NO and *NOH for p-CNTs, with ΔG values of 1.27 and 0.64 eV, respectively, which are much higher than those for W-CCNs (0.16 and 0.02 eV). This suggests that W-CCNs reduce the energy barriers for these hydrogenation steps (Figure 4h,i). This synergy between different defect sites created an environment conducive to multi-step NO_3_RR. The unique structure of W-CCNs, with pristine CNTs as the core and defect-doped outer layers, not only enhanced the adsorption of NO_3_^−^, but also facilitated the generation and utilization of *H, leading to improved NH_3_ production. Additionally, the study emphasized that the integration of multiple defect sites within a hierarchical structure introduced a novel operational principle, enabling C-MFECs to excel in various complex reaction systems.

### 4.2. Active Hydrogen Regulation

Active atomic hydrogen (H*) plays a pivotal role in the NO_3_RR process, as it directly participates in the hydrogenation of nitrate species, significantly enhancing the catalytic efficiency and selectivity towards ammonia production. Carbon-based materials, due to their tunable structure and surface properties, can be engineered to effectively modulate the generation and distribution of active atomic hydrogen. Strategies such as defect engineering, surface functionalization, and the introduction of metal nanoparticles on carbon substrates have been explored to optimize atomic hydrogen activation.

**Figure 5:**
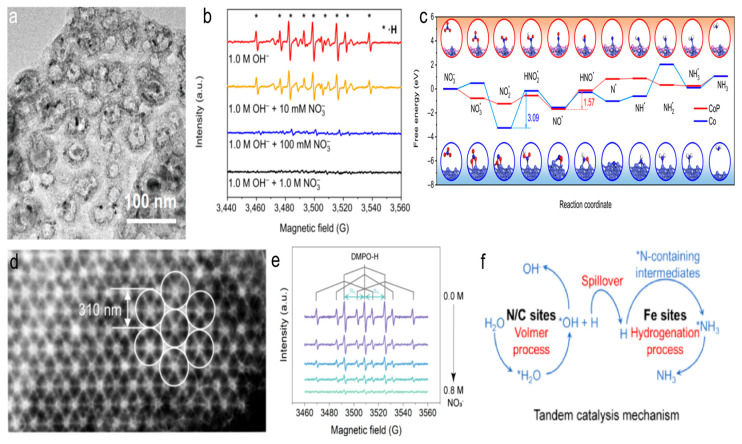
(**a**) STEM image of CoP-CNS. (**b**) ESR spectra of the CoP-CNS-catalyzed NO_3_RR solutions with different c NO_3_^−^ using DMPO as the radical trapping reagent. (**c**) Gibbs free energy diagram along the minimum energy pathway, along with the adsorption configurations of various intermediates formed during NO_3_RR. Reproduced from ref. [58] copyright (2022), with permission from Springer Nature. (**d**) HAADF-STEM images of the high-density metal/PNC. (**e**) ESR spectra of high-density Fe_1_/PNC with the NO_3_^−^ concentration increased from 0 to 0.8 M. (**f**) Schematic diagram of the tandem catalysis mechanism. Reproduced from ref. [60] copyright (2024), with permission from the American Chemical Society.

One study found that the high NH_3_ yield and Faradaic efficiency were primarily driven by the dynamic balance between the production of H* on CoP and its rapid consumption by nitrogen intermediates (Figure 5a–c) [58]. The CoP-CNS catalyst, with its unique structure, effectively balanced the production and consumption of H*, leading to superior electrocatalytic performance. This equilibrium ensured that H* was efficiently utilized by nitrogen-containing intermediates, promoting the conversion of NO_3_^−^ to NH_3_. Theoretical calculations revealed that the conversion from NO* to HNO* was the rate-determining step for CoP, with a smaller ΔG uphill of 1.57 eV, while for Co, the RDS was NO_2_* to HNO_2_* with a significantly higher ΔG of 3.09 eV. This was consistent with experimental observations and underscored the critical role of phosphatization in enhancing NO_3_RR kinetics. Additionally, the reaction pathway on the CoP surface was much smoother than on Co, indicating superior NO_3_RR activity on CoP-CNS. The research highlighted that maintaining this H* equilibrium was more effective for improving the NO_3_RR performance than merely suppressing water splitting. Likewise, the iron phosphide nanoparticles confined on a phosphorus-doped carbon substrates (FeP-PNC) catalyst, with its unique structure, effectively balanced the production and consumption of H*, leading to superior electrocatalytic performance [59]. FeP (011) improved the adsorption of NO_3_^−^ and NO_2_^−^, while the adjacent P-doped C sites facilitated water dissociation more efficiently than N-doped C reactive sites, supplying enough H* for the FeP (011) surface, where various hydrogenation steps of NO_3_^−^ take place. The synergy between P-doped C and FeP enabled the efficient use of H* by nitrogen-containing intermediates, driving the conversion of NO_3_^−^ to NH_3_.

Additionally, it has been observed that the high-density, atomically dispersed metal sites in metal-N-C catalysts can effectively activate nearby N/C sites, thereby enhancing water adsorption and dissociation [60,61]. This provided sufficient H* to the metal sites for nitrate ammoniation. The research highlighted that the high-density Fe in Fe_1_/PNC could more effectively activate the adjacent N/C sites compared to low-density counterparts. This activation improved water adsorption/dissociation and supplied sufficient active hydrogen to the Fe sites, thereby promoting the conversion of NO_3_^−^ to NH_3_ (Figure 5d–f). The findings provided valuable insights into the design of carbon-based catalysts for efficient electrochemical NO_3_RR, emphasizing the importance of H* management in catalytic systems.

### 4.3. Suppressing Competing Hydrogen Evolution Reaction

**Figure 6:**
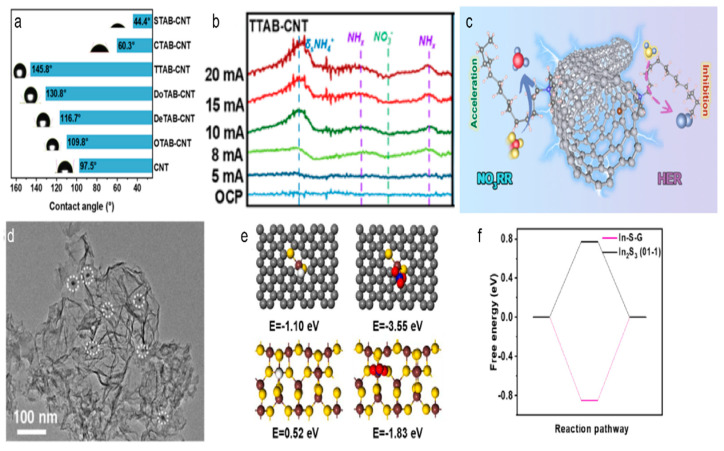
(**a**) Contact angle of the CNT modified by different quaternary ammonium surfactants. (**b**) Potential-dependent operando FTIR spectra of TTAB-CNT. (**c**) The proposed NO_3_RR and HER process on TTAB-CNT. Reproduced from ref. [64] copyright (2024), with permission from the American Chemical Society. (**d**) TEM image of the as prepared In-S-G. (**e**) The adsorption energy of H and NO_3_^−^ on the surface of In-S-G and In_2_S_3_(01-1). (**f**) The calculated Gibbs free energy of H* adsorption on the surface of In-S-G and In_2_S_3_(01-1). Reproduced from ref. [65] copyright (2021), with permission from Elsevier.

In the process of NO_3_RR, the efficient generation and control of H* is essential. However, the HER often competes with NO_3_RR, leading to reduced ammonia production. To enhance nitrate reduction efficiency, it is essential to suppress HER. One effective strategy involves the design of hydrophobic carbon surfaces [62,63]. By modifying the carbon material to enhance its hydrophobicity, the availability of H^+^ for the HER can be minimized, thus promoting selective nitrate reduction. For instance, a study by Liu et al. showed that trimethyltetradecylammonium bromide, with its moderate alkyl chain length, formed a highly hydrophobic interface on CNTs, resulting in about 87% selectivity for ammonia production [64]. This hydrophobic surface effectively suppressed the HER, which competed with nitrate reduction. In-depth mechanistic studies using operando Fourier transform infrared spectroscopy and online differential electrochemical mass spectrometry showed that the hydrophobic modification of CNTs enhanced the direct electron transfer pathway, promoting the reduction of nitrate to ammonia. The hydrophobic interface reduced the accessibility of protons at the electrode surface, thereby inhibiting HER, while the positively charged surfactant layer on CNTs favored the adsorption and enrichment of nitrate anions through electrostatic attraction (Figure 6a–c).

Another effective strategy to suppress HER is the incorporation of metal nanoparticles or doping heteroatom in carbon materials, which can influence molecular adsorption, thereby improving the Faradaic efficiency and selectivity towards ammonia. Lei et al. reported that indium incorporated in a sulfur-doped graphene (I-S-G) catalyst could achieve an ammonia production rate of 220 mmol gcat^−1^ h^−1^ and a Faradaic efficiency of about 75% at −0.5 V vs. RHE [65]. The abundant active sites and the hybrid electronic structure of the In species in the catalyst were crucial for promoting NO_3_RR and suppressing HER. The In-S-G catalyst showed a higher overpotential for HER, indicating efficient suppression of hydrogen production. DFT calculations revealed that the unique electronic structure of In-S-G could enable nitrate adsorption and activation while inhibiting H_2_ production, thus boosting the selective electroreduction of nitrate to NH_3_ (Figure 6d–f).

### 4.4. Tuning the Reaction Pathway

Carbon-based catalysts play a pivotal role in regulating reaction pathways in the NO_3_RR. These catalysts not only enhance the efficiency of the reduction process, but also play a crucial role in directing the formation of desired products. By modulating the electronic structure and surface characteristics of the catalyst, they can promote selective nitrate reduction, ensuring optimal reaction conditions and minimizing side reactions.

**Figure 7:**
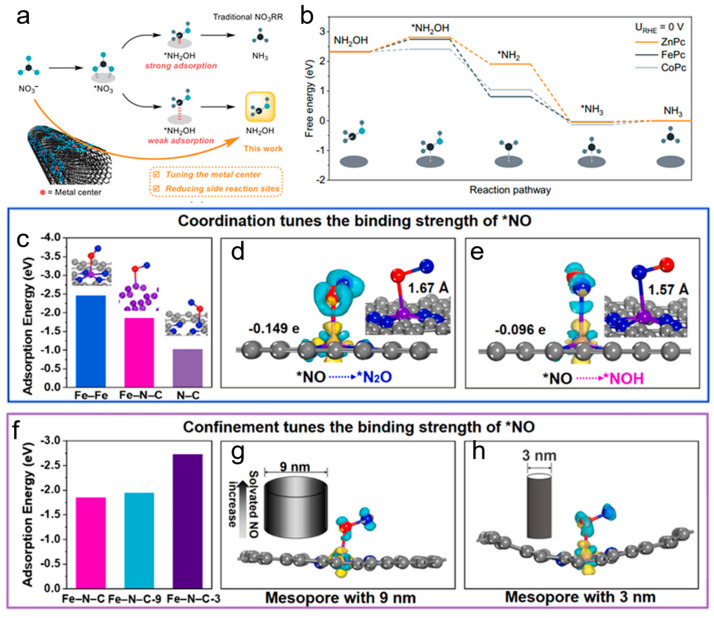
(**a**) Selective NH_2_OH production from nitrate using metal phthalocyanine electrocatalysts. (**b**) Free energy diagram for the NH_2_OHRR on different MPcs at 0 V vs. RHE. Reproduced from ref. [66] copyright (2024), with permission from Springer Nature. (**c**) Reaction energies of *NO adsorption on Fe bulk, Fe-N, and N-C in Fe-N-C. Charge density difference diagrams of *NO on Fe-N-C during the (**d**) N_2_ generation pathway (*NO to *N_2_O) and (**e**) NH_3_ formation pathway (*NO to *NOH), with insets showing the corresponding elongation of the N-O bond. (**f**) Reaction energies and charge density difference diagrams of *NO adsorption on Fe-N-C9 (Me-so-FeN-C with a 9 nm pore size) and Fe-N-C-3 with a 3 nm pore size (**g**,**h**). Molecular model: N in blue, O in red, Fe in purple, and C in gray. Reproduced from ref. [67] copyright (2023), with permission from Elsevier.

One study demonstrated that zinc phthalocyanines (ZnPc) supported on carbon nanotube (CNT) substrates could effectively suppress the further reduction of hydroxylamine (NH_2_OH) [66]. By tuning the intrinsic activity of ZnPc and covering the CNT surface with ZnPc molecules to block side reaction sites, the catalyst achieved a Faradaic efficiency of 53 ± 1.7% for NH_2_OH with a partial current density exceeding 270 mA cm^−2^ and a turnover frequency of 7.5 ± 0.2 s^−1^. The free energy diagrams revealed that the NH_2_OH reduction reaction process encounters an initial energy barrier during NH_2_OH adsorption, after which the process proceeds through several downhill steps. CoPc displayed a low NH_2_OH adsorption energy barrier of 0.09 eV, promoting further NH_2_OH reduction. In contrast, ZnPc and FePc exhibited higher barriers, with ZnPc showing the highest at 0.49 eV, indicating that it had lower activity for NH_2_OH reduction and was thus more selective for NH_2_OH production in NO_3_RR (Figure 7a,b). This shows how carbon-based catalysts can be designed to selectively produce high-value intermediates like NH_2_OH by controlling reaction pathways and suppressing competing reactions.

Another study emphasized the critical role of carbon-based catalysts in steering reaction pathways towards the selective production of NH_3_ or N_2_. A novel Fe single-atom catalyst coordinated with N on an ordered mesoporous carbon framework (Meso-Fe-N-C) was developed, demonstrating excellent electrocatalytic denitrification performance [67]. The N coordination and mesoporous structure tuned the electronic structure of the Fe site, leading to a modest adsorption binding strength with key intermediates. Theoretical calculations indicated that the Gibbs free energy for *N_2_O generation (ΔG(*N_2_O) = −0.30 eV) was significantly lower than that for *NOH formation (ΔG(*NOH) = 0.41 eV), suggesting that the 5-electron transfer leading to N_2_ production was more thermodynamically favorable than the 8-electron transfer required for NH_3_/NH_4_^+^ formation. Moreover, the mesoporous carbon not only enhanced the adsorption and transfer of NO_3_^−^ but also confined reaction intermediates, promoting N_2_ formation. (Figure 7c–h) The research underscored how precise control of catalyst architecture, from microscopic electron distribution to macroscopic physical structure, could effectively steer reaction pathways toward desired products.

### 4.5. Enhancing Reaction Durability

Enhancing the durability of carbon-based catalysts is crucial for the efficient and sustainable NO_3_RR. The long-term stability of the catalyst ensures consistent performance, especially under the harsh electrochemical conditions of the reaction. Carbon materials can improve catalyst longevity through strategies such as the incorporation of robust metal–carbon interactions, the optimization of surface functionalization, and defect engineering. These modifications help prevent catalyst deactivation due to corrosion or leaching, thereby maintaining high catalytic activity and selectivity over extended reaction cycles. By enhancing the stability of carbon-based catalysts, the overall efficiency and practicality of NO_3_RR are significantly improved.

**Figure 8:**
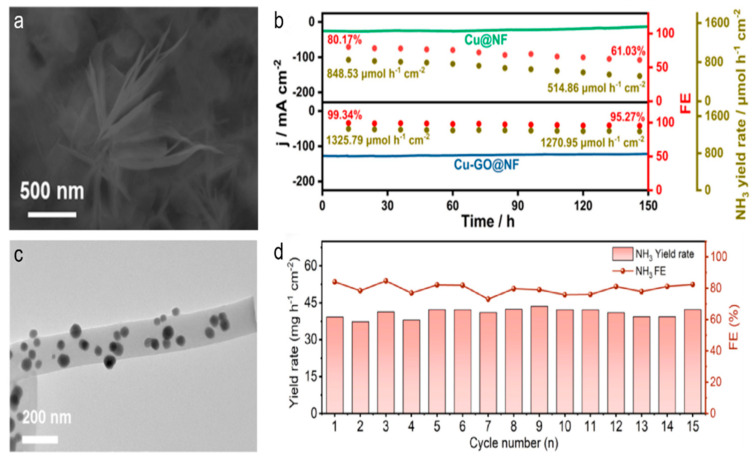
(**a**) SEM image of Cu-GO@NF. (**b**) The catalytic activity and stability of Cu-GO@NF and Cu@NF catalysts were evaluated in a flow cell with a 1 M KOH solution containing 200 ppm NO_3_^−^-N at −0.13 V. Reproduced from ref. [68] copyright (2025), with permission from Elsevier. (**c**) TEM image of CoSn-CNF. (**d**) The stability test at −0.6 V on a CoSn-CNF catalyst. Reproduced from ref. [69] copyright (2024), with permission from Elsevier.

The enhancement of catalyst stability and NO_3_RR performance through metal–carbon interactions in carbon-based catalysts has been highlighted [68,69,70,71]. The Cu-GO@NF sample, fabricated via a low-temperature calcination method, leveraged the synergistic effects of Cu and Ni metals with GO [68]. Crucially, the hydrated cation–π interactions between GO and the metal components (Cu and Ni) significantly improved the stability of the Cu-GO@NF electrocatalyst. During a 150 h continuous reaction, the Cu and Ni contents in the Cu-GO@NF decreased by just 3.3% and 4.5%, respectively, in contrast to the substantial reduction of 34.0% and 51.9% in the Cu@NF sample without GO modification. This indicated that the introduction of GO effectively shielded the metal components from loss and degradation, thereby maintaining the catalyst’s stability and performance (Figure 8a,b). Overall, the metal–carbon interactions in the Cu-GO@NF catalyst played a vital role in enhancing both stability and activity for efficient electrochemical nitrate reduction to ammonia.

Additionally, the confined spaces within nanostructured carbon materials, such as carbon nanotubes (CNTs), have been shown to further promote the stability of nitrate reduction [72,73,74]. For example, the carbon nanofiber (CNF) served as a support that prevented the leaching and agglomeration of metal particles, ensuring exceptional cycling performance [69]. The CoSn-CNF catalyst, in particular, demonstrated remarkable stability and high activity. After reaction, CoSn-CNF retained its fibrous architecture and the CoSn alloy particles remained evenly distributed without aggregation or leaching. The CNF structure provided a robust environment for the CoSn alloy nanoparticles (Figure 8c,d). The combination of the CNF support and the electronic structure modulation of the bimetallic alloy leads to a highly efficient and stable electrocatalyst for ammonia production from nitrate reduction.

As illustrated above, the NO_3_RR can be divided into three key steps: reactant adsorption/activation, proton-coupled electron transfer, and product desorption. Based on these three key steps, we have summarized the structural features and properties of carbon materials, along with their catalytic roles in facilitating these major stages of the reaction process. The summary is provided in the table below (Table 2).

Moreover, Table 3 provides a direct comparison of the performance of various carbon-based catalysts in the reduction of NO_3_RR to NH_3_. The table summarizes key performance metrics such as ammonia yield, Faradaic efficiency, and selectivity for each catalyst, offering a clear overview of their comparative effectiveness in the NO_3_RR process. This comparison highlights the strengths and potential areas for improvement of each material in ammonia production.

## 5. Conclusions, Challenges, and Perspectives

The electrochemical NO_3_RR using carbon-based materials has emerged as a promising and sustainable technology for environmental remediation and nitrogen recovery. Carbon materials offer several key advantages in this field, particularly their structural diversity and sustainability. Their unique properties can be fine-tuned through strategies such as doping, functionalization, and defect engineering, allowing the design of catalysts with enhanced selectivity, high surface area, and improved conductivity. This adaptability makes carbon-based materials highly effective for nitrate reduction reactions. Additionally, their sustainability—especially when derived from renewable sources like biomass—provides significant environmental benefits compared to traditional metal-based catalysts.

The integration of material design, mechanism analysis, and engineering application is crucial for advancing the performance and practical deployment of carbon-based catalysts for NO_3_RR. Innovative material design must be coupled with a deep understanding of the reaction mechanisms at the atomic and molecular levels to optimize the catalytic activity and selectivity. Mechanism analysis helps to identify key intermediates and the roles of different active sites, which can inform the rational design of catalysts with higher efficiency and stability. Finally, translating these advances into engineering applications requires addressing challenges such as scalability, catalyst stability under operational conditions, and integration with existing industrial systems. This full-chain innovation approach is essential to realize the potential of carbon-based catalysts for nitrate reduction in real-world applications. Although carbon-based materials show promising potential in NO_3_RR, several challenges persist; however, certain perspectives offer hope for overcoming them.

(1) Toxicity of chloride and organic contaminants in real wastewater. One of the major challenges in utilizing carbon catalysts for nitrate reduction in real-world wastewater is the presence of chloride ions and organic contaminants. These impurities can poison the catalyst by blocking active sites or altering the electronic properties of the carbon material, thus reducing its efficiency and lifetime. Addressing this issue requires the development of robust carbon catalysts that can tolerate complex wastewater compositions without significant degradation in performance.

(2) Corrosion and structural collapse at high current densities. At higher current densities, carbon materials are susceptible to corrosion and structural collapse. This phenomenon occurs due to the electrochemical environment under high current loads, which can lead to the degradation of the carbon structure and loss of catalytic activity. Therefore, it is critical to design carbon materials that can withstand the harsh conditions of high current densities without compromising their structural integrity and catalytic performance.

(3) Integrating multi-scale characterization. While ex situ analysis and DFT calculations have provided valuable mechanistic insights into carbon-catalyzed nitrate reduction, real-time monitoring of dynamic processes plays a critical role in connecting these predictions to actual catalytic behavior. These in situ approaches offer unique advantages, such as resolving carbon-specific processes like dopant transformation under applied potentials, intermediate transport within confined pores, and electric double-layer restructuring. Establishing standardized protocols for such real-time analyses will not only enhance mechanistic understanding, but also accelerate the rational design of next-generation catalysts.

(4) Machine learning-guided rational design. The application of machine learning techniques for the rational design of carbon materials offers the potential to accelerate the discovery of new catalysts with optimized properties. By using computational models and high-throughput screening, machine learning can help identify key descriptors, such as doping types and defect densities, that are critical for enhancing nitrate reduction performance. This approach can lead to more efficient and cost-effective materials design.

(5) Carbon neutrality through biomass-derived catalysts. The drive towards carbon neutrality presents a significant opportunity for the development of biomass-derived carbon catalysts. These materials, derived from renewable organic sources, can be synthesized with controlled properties, such as varying levels of doping or defect density, to optimize catalytic activity. Biomass-derived catalysts could play a crucial role in achieving a sustainable and circular carbon economy by providing an alternative to traditional fossil fuel-based catalysts.

(6) Integrated systems development. A promising direction for the future is the development of integrated systems that combine NO_3_RR with other technologies, such as ammonia fuel cells. By coupling nitrate reduction with ammonia electrolysis or fuel cells, it is possible to create systems that not only produce green ammonia, but also generate electricity or store energy. These integrated systems could significantly improve the overall efficiency and sustainability of nitrogen waste treatment, green ammonia production, and hydrogen energy storage.

## Figures and Tables

**Table 1:** Critical trade-off analysis of carbon material platforms for NO_3_RR.

Material	Advantages	Disadvantages	Key Trade-Offs
**Graphene/GO**	Ultra-high conductivity Tunable oxygen functional groups Atomic-level thickness enabling full active site exposure	Limited mass loading capacity Severe aggregation in aqueous electrolytes Prohibitively high cost at industrial scale	Conductivity vs. stability: reduced GO enhances conductivity but depletes catalytically active oxygen groups
**CNTs**	1D electron highway accelerates charge transfer Nanoconfinement effects stabilizing intermediates Exceptional mechanical robustness	Insufficient mesoporosity restricting diffusion Poor dispersion requiring functionalization	Confinement vs. mass transport: tubular confinement enhances adsorption but impedes N_3_O^−^ diffusion
**Porous Carbons**	Ultra-high surface area maximizing active sites Tunable pore hierarchy optimizing mass transfer Low-cost precursors (biomass/polymers)	Poor intrinsic conductivity Pore flooding in gas-diffusion electrodes Heterogeneous active site distribution	Surface area vs. conductivity: higher surface area increases sites but compromises charge transfer efficiency
**Heteroatom-Doped Carbons**	Precise electronic structure tuning Designable Lewis acid/base sites Synergistic co-doping effects	Dopant leaching during long-term operation Complex synthesis requiring strict control Limited doping depth	Doping precision vs. stability: high-temperature annealing improves stability but depletes surface dopants
**Defective Carbons**	Abundant edge sites for proton-coupled reactions Strain-induced activation lowers energy barriers Intrinsic proton relay capability	Low crystallinity reducing conductivity Uncontrollable defect types High vulnerability to electrochemical oxidation	Defect density vs. structural integrity: more defects enhance activity but accelerate carbon corrosion
**Metal-Loaded Carbons**	Atomic-scale activation for N-O bond cleavage Tunable metal coordination environments	Critical metal leaching/dissolution issues Competing H_2_ generation High cost for precious metal loading	Metal activity vs. selectivity: increased metal loading boosts conversion but promotes H_2_ byproduct formation

**Table 2:** Carbon material design for key steps in NO_3_RR.

Dominant Process	Critical Carbon Properties	Optimal Material Types	Mechanistic Function
NO_3_^−^ adsorption/ activation	Surface charge density Lewis acidic sites Conductive networks	Heteroatom-doped carbon Carbon-supported metal Graphene oxide	Enhances NO_3_^−^ adsorption energy Reduces N-O bond cleavage barrier Accelerates interfacial electron injection
Proton–electron transfer	Local hydrophilicity Proton-relay groups Buffered microenvironment	Porous carbon Heteroatom-doped carbon Defective carbon	Concentrates H^+^ Reduces activation energy Alters reaction pathway
NH_3_ desorption	Intermediate adsorption strength Hydrophobic microdomains Electric double-layer modulation	Heteroatom-doped carbon Graphene/CNTs Strained carbon lattices	Weakens NH_2_ adsorption Lowers NH_3_ desorption barrier Prevents catalyst poisoning

**Table 3:** Summary of performance of various catalysts for reduction of electrochemical NO_3_RR to NH_3_.

Catalyst	Electrolyte, Potential	NH_3_ Yield Rate	Faraday Efficiency	Selectivity	Reference
**Nitrogen-doped carbon aerogel**	0.1 M KOH and 0.1 M KNO_3_, −0.7 V vs. RHE	1.33 mg NH_3_ h^−1^ cm^−2^	95%	/	[30]
**B/O-CNNTs**	0.1 M KNO_3_ and 0.1 M Na_2_SO_4_, −1.1 V vs. RHE	22.56 μg h^−1^ mg^−1^	82.49%	/	[37]
**Amorphous graphene**	1 M NaNO_3_, −0.93 V vs. RHE	2859 μg h^−1^ cm^−2^	≈100%	>70%	[40]
**Fe N/P C**	0.1 M KOH and 0.1 M KNO_3_, −0.4 V vs. RHE	17,980 μg h^−1^ mg_cat_^−1^	90.3%	/	[44]
**Cu-PPy**	0.5 M H_2_SO_4_ and 0.1 M KNO_3_, −0.3 V vs. RHE	0.55 mmol h^−1^ cm^−2^	96.0%	99.99%	[45]
**CoO NC/graphene**	1 M KOH and 1 M KNO_3_, −400 mA cm^−2^	25.63 mol g^−1^ h^−1^	>98%	>98%	[47]
**FeCo@CNFs-Fe/N**	0.1 M NaOH and 0.1 M NaNO_3_, −0.41 V vs. RHE	498.18 μmol g^−1^ h^−1^	87%	/	[51]
**RuCu DAs/NGA**	0.1 M KOH and 0.1 M KNO_3_, −0.4 V vs. RHE	3.1 mg h^−1^ cm^−2^	95.7%	/	[52]
**Fe@N10-C**	0.5 M Na_2_SO_4_ and 500 mg L^−1^ NaNO_3_, −0.75 V vs. RHE	2647.7 μg h^−1^ cm^−2^	91.8%	~100%	[56]
**Carbon coaxial nanocables**	1 M KOH and 0.1 M KNO_3_, −0.5 V vs. RHE	88.1 g h^−1^ g^−1^	~65%	/	[57]
**CoP-CNS**	1 M OH^−^ and 1 M NO_3_^−^, −1.03 V vs. RHE	3.09 ± 0.10 mmol h^−1^ cm^−2^	~90%	/	[58]
**Fe_1_/PNC**	1 M KOH and 100 mM NO_3_^−^, −0.57 V	7.95 ± 0.22 mol h^−1^ g^−1^	90.44 ± 0.57%	/	[60]
**Hydrophobic modified CNT**	0.1 M NO_3_^−^ and 50 mg-N L^−1^ NaNO_3_, 10 mA cm^−2^	/	/	~87%	[64]
**In-S-G**	1 M KOH and 0.1 M KNO_3_, −0.5 V vs. RHE	220 mmol h^−1^ g^−1^	75%	/	[65]
**Ni@TiN/CNT**	0.5 M K_2_SO_4_ and 0.05 M KNO_2_, −0.7 V vs. RHE	15.6 mg h^−1^ mg^−1^	95.6%	/	[71]
**Cu-GO@NF**	1 M KOH and 200 mg L^−1^ NO_3_^−^-N, −0.13 V vs. RHE	/	99.51%	95.03%	[68]
**CoSn-CNF**	1 M KOH with 0.1 M KNO_3_, −0.6 V vs. RHE	42.20 mg h^−1^ cm^−2^	81.5%	/	[69]
**FeN_4_/Fe_4_@mCNR**	1 M KOH and 0.1 M NO_3_^−^, −0.3 V vs. RHE	5.52 mg h^−1^ cm^−2^	98.6%	/	[75]

## Data Availability

No new data were created or analyzed in this study. Data sharing is not applicable to this article.
